# Classification of degenerative parkinsonism subtypes by support-vector-machine analysis and striatal ^123^I-FP-CIT indices

**DOI:** 10.1007/s00415-019-09330-z

**Published:** 2019-04-29

**Authors:** Nicolas Nicastro, Jennifer Wegrzyk, Maria Giulia Preti, Vanessa Fleury, Dimitri Van de Ville, Valentina Garibotto, Pierre R. Burkhard

**Affiliations:** 1 Department of Psychiatry, University of Cambridge, Hills Road, Cambridge, CB2 0SP UK; 2 Division of Neurology, Geneva University Hospitals, Geneva, Switzerland; 3 Center for Neuroprosthetics, Institute of Bioengineering, EPFL, Lausanne, Switzerland; 4 Department of Radiology and Medical Informatics, Geneva University Hospitals, Geneva, Switzerland; 5 Division of Nuclear Medicine and Molecular Imaging, Geneva University Hospitals, Geneva, Switzerland

**Keywords:** Parkinson’s disease, Degenerative parkinsonism, SPECT, Classification

## Abstract

**Objectives:**

To provide an automated classification method for degenerative parkinsonian syndromes (PS) based on semiquantitative ^123^I-FP-CIT SPECT striatal indices and support-vector-machine (SVM) analysis.

**Methods:**

^123^I-FP-CIT SPECT was performed at a single-center level on 370 individuals with PS, including 280 patients with Parkinson’s disease (PD), 21 with multiple system atrophy-parkinsonian type (MSA-P), 41 with progressive supranuclear palsy (PSP) and 28 with corticobasal syndrome (CBS) (mean age 70.3 years, 47% female, mean disease duration at scan 1.4 year), as well as 208 age- and gender-matched control subjects. Striatal volumes-of-interest (VOIs) uptake, VOIs asymmetry indices (AIs) and caudate/putamen (C/P) ratio were used as input for SVM individual classification using fivefold cross-validation.

**Results:**

Univariate analyses showed significantly lower VOIs uptake, higher striatal AI and C/P ratio for each PS in comparison to controls (all *p* < 0.001). Among PS, higher degree of striatal impairment was observed in MSA-P and PSP, while CBS showed moderate uptake reduction and higher AI. Binary SVM classification showed 92.9% accuracy in distinguishing PS from controls. Classification based on each binary combination of PS ranged 62.9–83.7% accuracy with the most satisfactory results when separating CBS from the other PS. Sensitivity and specificity values were high and balanced ranging from 60 to 80% for all analyses with > 70% accuracy. Overall, striatal AI and C/P ratio on the more affected side had the highest weighting factors.

**Conclusion:**

Semiquantitative ^123^I-FP-CIT SPECT striatal evaluation combined with SVM represents a promising approach to disentangle PD from non-degenerative conditions and from atypical PS at the early stage.

## Introduction

Parkinson Disease (PD) is the most common neurodegenerative movement disorder in the general population and accounts for 65% of all parkinsonian syndromes (PS) [1]. It is characterized by progressive akinesia in association with rest tremor and muscular rigidity as well as an excellent response to levodopa therapy [2]. Clinical diagnosis is not always straightforward and distinction from atypical parkinsonian syndromes (APS) can be particularly challenging in the early phase. In fact, autopsy-based studies have shown that diagnostic accuracy for PD does not exceed 79.6% even when clinical diagnosis is performed by movement disorders experts, or 82.7% when using stringent clinical criteria for PD [3]. Misclassification involves other degenerative conditions—i.e., multiple system atrophy (MSA), progressive supranuclear palsy (PSP) and corticobasal degeneration (CBD). Compared to PD, APS are characterized by additional clinical features besides parkinsonism (cerebellar or pyramidal signs, postural instability or apraxia), a moderate or transient response to levodopa and a worse prognosis with a mean life expectancy of 7–10 years from disease onset [4, 5]. The issue of PD clinical misdiagnosis has virtually not improved over the last 25 years, and various biomarkers, notably structural and metabolic imaging, have been examined as potential aids to clinical diagnosis and, to a lesser extent, to separate the different forms of degenerative PS [6]. As yet, however, only a few of the proposed biomarkers have proved robust enough to be included in the new MDS criteria for PD—olfactory loss and cardiac sympathetic denervation as supportive criteria, and a normal presynaptic dopaminergic system as exclusion criterion [2].

Presynaptic dopaminergic pathways integrity can be assessed with various compounds, ^123^I-FP-CIT (ioflupane) being the most widely used due to its fast kinetics, selectivity to dopamine transporters (DaT), SPECT scan availability, and its binding not being affected by concurrent dopaminergic treatment [7]. While visual and semiquantitative assessments have shown excellent sensitivity and specificity (resp. 97 and 100%) in differentiating early clinically diagnosed PD subjects (showing a reduced striatal uptake) from non-degenerative cases, similar observations could not be drawn for the distinction of PD and APS [8–11]. In fact, there is a large consensus among experts that DaT SPECT is not able to separate PD from APS, although this assumption has not been extensively studied. In fact, recent publications have shown data partly challenging this consensus. At the group level, PD subjects usually harbor an asymmetric rostrocaudal progression of DaT denervation, with a preservation of caudate nucleus uptake in the early phase, whereas MSA-parkinsonism (MSA-P) and PSP usually show a more global uptake impairment [12]. In patients with corticobasal syndrome (CBS), a highly asymmetric and moderate uptake alteration has been described [13], with several cases presenting a virtually normal SPECT [8, 14]. However, individual detection of subjects with degenerative PS based on molecular imaging remains elusive.

Support vector machine (SVM) is a widely used pattern-recognition method that learns to assign labels to feature vectors [15] and has been used increasingly for automated classification in a variety of medical applications (for a review, see [16]). SVMs have been applied to distinguish PD from controls (CTL) or APS based on usually complex and time-consuming processing of various imaging techniques—e.g., DaT SPECT, postsynaptic dopamine PET, diffusion tensor imaging (DTI) or voxel-based morphometry (VBM) MRI imaging with accuracies ranging from 70 to 100% [17–25]. Thanks to multivariate pattern-recognition analyses of ^123^I-FP-CIT SPECT, we recently observed a differential pattern of uptake alteration between PD and APS; i.e., a more severe caudate nucleus impairment in MSA and PSP, and a relative preservation of putaminal uptake in CBS compared to PD [26].

We here propose to reappraise the issue of distinguishing the various forms of degenerative PS by means of an SVM classification solely based on semiquantitative ^123^I-FP-CIT SPECT striatal parameters, the most commonly used output to support diagnosis in clinical practice, obtained from a large, single-center cohort of subjects with idiopathic PD, MSA-P, PSP and CBS in the early stage of disease (< 3 years) and with the same SPECT protocol.

## Methods

### Patients inclusion

The present study was conducted in compliance with the declaration of Helsinki and was approved by the canton of Geneva Ethics Committee (CER 12-006R). As a retrospective study evaluating SPECT imaging performed in the diagnostic setting, written consent from subjects was waived. ^123^I-FP-CIT SPECT was performed between October 2003 and October 2016, and the following clinical data were collected: sex, diagnosis, age at scan, disease duration at scan. Whenever a subject had two or several scans, only the first one was considered. Besides, only SPECT scans acquired on the same scanner and with the same protocol were included. All subjects with sporadic and idiopathic PD, MSA-P, PSP and CBS were classified according to the most recent clinical diagnostic criteria [2, 4, 5, 27], with a disease duration < 3 years at scan and age > 40 years at disease presentation to exclude potentially genetic young-onset PD cases. Disease duration at scan was considered as the time between onset of motor symptoms and performing the SPECT scan. In addition, we included a CTL group, i.e., subjects with non-degenerative parkinsonism or tremor [essential tremor (ET), drug-induced parkinsonism (DIP) and psychogenic parkinsonism (PP)] associated with preserved DaT uptake based on both visual and semiquantitative assessments, and with identical restrictions regarding age (> 40 years) and disease duration at scan (< 3 years).

### SPECT imaging acquisition and reconstruction

**Fig. 1:**
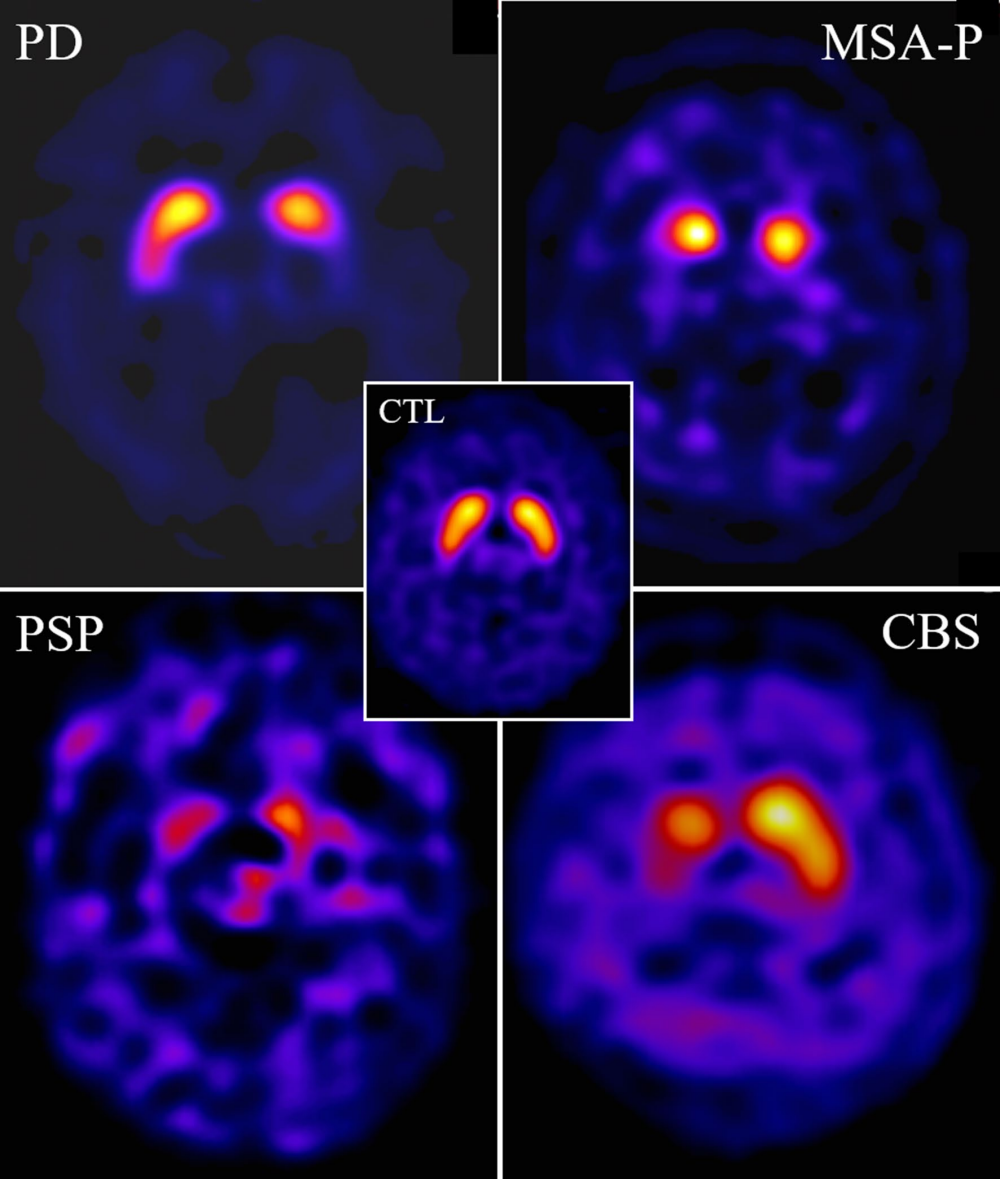
Illustrative examples of ^123^I-FP-CIT SPECT scan images (axial slices) performed within a year after disease onset in patients with autopsy-proven PD (M, 65 years old), MSA-P (M, 67), PSP (F, 60) and CBS (F, 67), compared to a 65-year-old control female patient with dystonic tremor (CTL)

Details on the acquisition of SPECT imaging are available in Ref. [28]. Illustrative examples of typical patterns of DaT SPECT alterations in patients with PD, MSA, PSP and CBD, and of CTL subjects are shown in Fig. 1.

### Semiquantitative SPECT assessment

The BRASS™ automated functional brain analysis software [29] was used to extract semiquantitative uptake values for the following volumes-of-interest (VOIs): right and left caudate nucleus (C), putamen (P), striatum (S) as well as occipital lobe which was used as the reference. Uptake values regarding S were obtained by geometrical interpolation with the following formula: ${\text{uptake}}\; ( {\text{S}})\, = \,({\text{uptake}}\; ( {\text{C}})\; \times \;{\text{volume}}\; ( {\text{C}})\, + \,{\text{uptake}}\; ( {\text{P}})\; \times \;{\text{volume}}\; ( {\text{P}}))/{\text{volume}}\; ( {\text{S}})$ with volume based on the Talairach atlas (in voxels). Therefore, the formula used to calculate S uptake was ((C × 524) + (P × 689))/1213. Asymmetry indices (AI) for each striatal VOI (C-AI, S-AI and P-AI) and C/P ratio on the most and least affected side have been calculated accordingly. For the following analyses, we used C, P and S uptake as well as C/P ratio considering the most and least affected side, based on semiquantitative uptake values.

### Support-vector-machine classification analysis

Due to the presence of strongly imbalanced classes as reflected by disease prevalence (notably PD vs. each APS subtype), the R-package “ROSE” (Version i2015) was applied to balance the dataset for binary classification using a smoothed bootstrap approach [30, 31]. The classification analysis was then performed using the “Classification Learner App” (MATLAB 2016b, MathWorks). A linear SVM classifier was applied to learn a discriminant function that would optimally separate the respective class labels. Each training model was based on the ^123^I-FP-CIT SPECT uptake of a set fourteen features: uptake on the more affected side (more), less affected side (less), mean uptake value (mean), and AI for each ROI, i.e., S, C and P; as well as the C/P ratio on the more affected [C/P ratio (more)] and the less affected side [C/P ratio (less)]. The classes included in the classification procedure were chosen according to clinical decision-making.

**Fig. 2:**
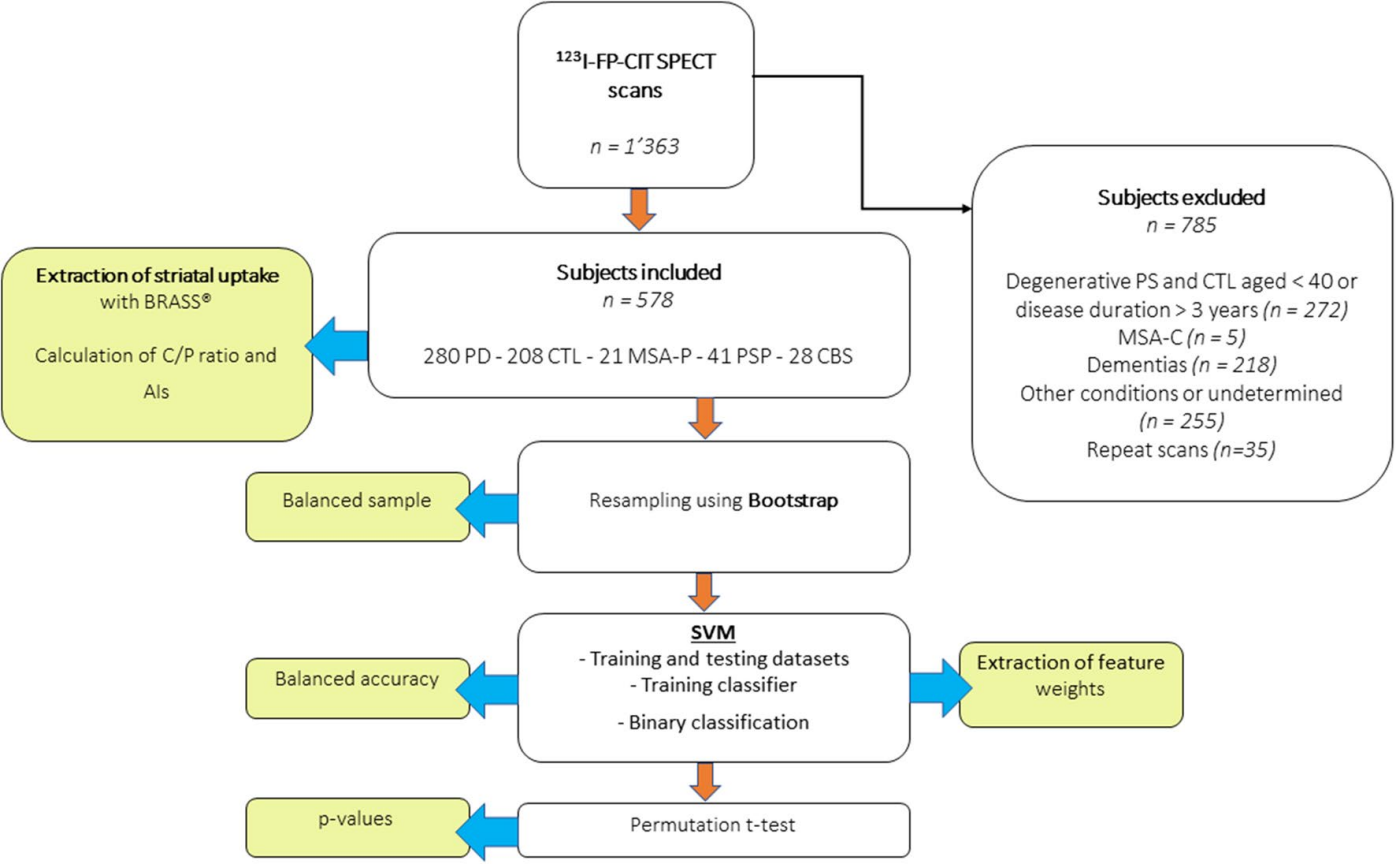
Flowchart of the study, summarizing patient inclusion, image processing and SVM analysis

Once the classifier trained, the code was exported to the MATLAB workspace and run using a custom-made script to extract the SVM weights (betas). For each model (i.e., all PS vs. CTL; PD vs. APS; each PS vs other PS; and all binary combination between PS), classification accuracy (Acc), specificity (Sp) and sensitivity (Sn) as well as the receiver operating characteristics-area under curve (ROC-AUC) were calculated using fivefold cross-validation. Acc measures the probability that a random patient would be correctly identified by the classifier. The statistical significance of the Acc measures was obtained by establishing its null distribution under random permutations (1000 times) of the class labels. Finally, we minimized the false-positive rate for APS by determining a high specificity of 0.95 for PD classification against other PS. We reported AUC measures as they reflect classification performance across every possible specificity–sensitivity trade-off and are not sensitive to sample distribution. A flowchart summarizing the different steps of subjects’ inclusion and SVM processing is available as Fig. 2.

### Statistics

Statistical analysis was performed with Stata software Version 14.2 (College Station, TX). Assessment of distribution for continuous variables was performed with Shapiro–Wilk test and visualization of histogram plots, followed by ANOVA or Kruskal–Wallis (KW) tests accordingly, and *t* test or Mann–Whitney *U* (MWU) test for two-group post hoc comparisons. Statistical significance was considered if *p* < 0.05. When assessing semiquantitative uptake between CTL and each PS, and among PD and APS, we used Bonferroni correction for each dependent variable (10 pairwise comparisons so *p* < 0.05/10 = 0.005). Values are usually reported as mean ± standard deviation (SD) (range). We also performed ROC curves with determination of cut-off points using the most significant univariate predictor and Youden index (best specificity/sensitivity trade-off) to design a tentative flowchart.

## Results

### Patients’ baseline characteristics

1363 subjects were scanned in our institution with the same SPECT acquisition protocol during the 13-year study period, among which 370 participants presented with a degenerative PS and fulfilled inclusion criteria (age ≥ 40, disease duration ≤ 3 years): 280 PD, 21 MSA-P, 41 PSP and 28 CBS subjects. As previously shown by our group, MSA-P and MSA-C have a very distinct dopamine SPECT pattern [32], so we decided not to include MSA-C subjects in further analysis as the MSA-C group was very small (*n* = 5). A total of 208 age-matched CTL subjects were also included. Mean follow-up was 4.4 ± 2.9 years (range 0.3–11.3). Baseline characteristics and VOIs uptake values for each group are available in Table 1.

**Table 1:** Baseline clinical characteristics of degenerative parkinsonism and control groups

	PD	MSA-P	PSP	CBS	Control	*P*val	*P*val
#	280	21	41	28	208	Degenerative PS and Control	Degenerative PS
Age (years)	69.8 ± 10.8 (42–92)	69.0 ± 11.0 (43–86)	72.7 ± 7.6 (44–85)	73.6 ± 7.8 (56–85)	69.9 ± 10.6 (42–91)	0.25^a^	0.14^a^
Disease duration (years)	1.3 ± 0.8 (0–3)	1.5 ± 0.8 (0.5–3)	1.6 ± 0.9 (0.3–3)	1.6 ± 0.7 (0.5–3)	NA		0.09^a^
Male/female ratio	1.12 (148/132)	1.1 (11/10)	1.05 (21/20)	1.15 (15/13)	0.66 (83/125)	0.07^b^	0.99^b^
S uptake	1.79 ± 0.64 (0.24–4.14)	1.35 ± 0.56 (0.49–3.17)	1.38 ± 0.60 (0.39–2.82)	1.96 ± 0.76 (0.20–4.04)	3.16 ± 0.55 (1.78–5.33)	All < 0.005^a^	
C uptake	2.25 ± 0.71 (0.31–4.63)	1.80 ± 0.61 (0.80–3.56)	1.70 ± 0.64 (0.48–3.17)	2.20 ± 0.84 (0.25–4.24)	3.37 ± 0.62 (1.55–6.05)		
P uptake	1.44 ± 0.64 (0.19–3.76)	1.01 ± 0.54 (0.14–2.88)	1.13 ± 0.60 (0.17–2.59)	1.78 ± 0.75 (0.17–3.89)	3.01 ± 0.54 (1.44–4.79)		
C/P ratio (most affected side)	1.86 ± 0.67 (0.47–6.73)	2.35 ± 1.37 (1.26–6.79)	1.72 ± 0.46 (0.79–2.82)	1.37 ± 0.41 (0.72–2.65)	1.13 ± 0.15 (0.71–1.75)		
Striatal AI	19.8 ± 14.8 (0.4–73.4)	19.7 ± 18.0 (0.4–63.2)	13.7 ± 10.9 (0.1–44.5)	31.3 ± 29.4 (1.6–150.6)	4.8 ± 3.7 (0.0–19.2)		

Mean (left + right) VOIs uptake, C/P ratio and striatal AI are also shown. Results are expressed Mean ± SD (range)

Statistical analysis: ^a^Kruskall Wallis test, ^b^Chi-squared test

### ^123^I-FP-CIT SPECT semiquantitative results

**Fig. 3:**
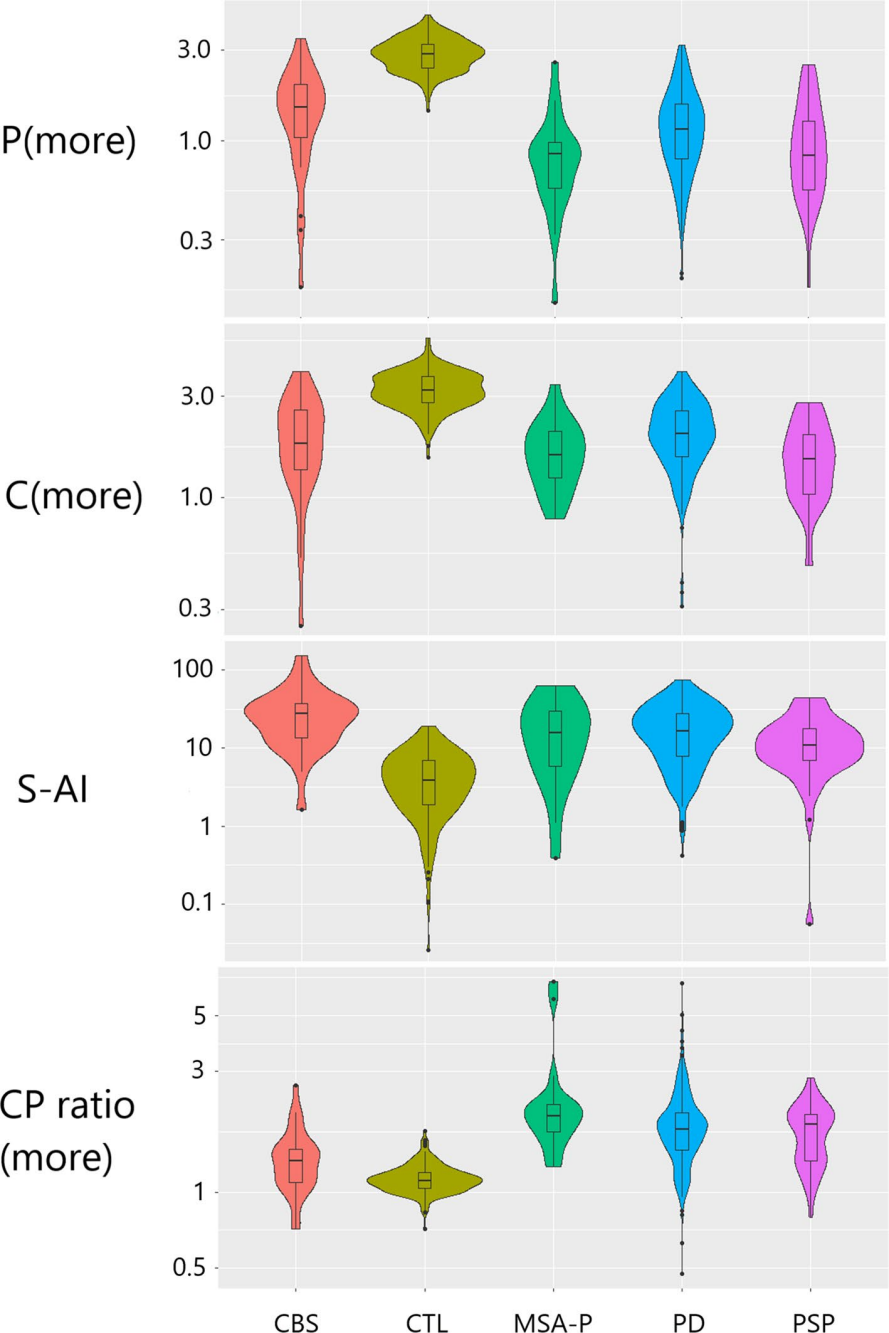
Violin plots of representative striatal outcomes [C(more), P(more), S-AI, C/P(more)] for the different groups included in the study. Outcome values are shown on a logarithmic scale

As expected, univariate analyses showed that, in comparison to CTL, each PS group (PD, MSA-P, PSP and CBS) had higher S-AI and C/P (more) (*p* < 0.001, MWU test) and significantly lower mean S, C and P uptake values (*p* < 0.0001) when using age-adjusted previously established local reference limits [28] **(**Fig. 3**)**.

Among degenerative conditions, head-to-head comparisons showed that PD subjects exhibited significantly higher mean uptake in the S, C and P compared to MSA-P (all *p* < 0.002) and PSP (all *p* < 0.005) and lower P uptake compared to CBS (*p* < 0.005). CBS subjects also exhibited higher S and P uptake compared to MSA-P (all *p* < 0.0005) and to PSP (all *p* < 0.001).

Regarding CBS, S-AI was significantly higher than for PSP (*p* = 0.0004) and C/P ratio (more) was lower than for all other PS (all *p* < 0.005).

### Linear SVM binary classification

Binary classification of all PS versus Controls showed excellent results (Sp 96.7%, Sn 89.5%, Acc 92.9%), with S-AI, S uptake and C/P ratio (more) being the strongest predictors (Table 2).

**Table 2:** Classification performance of the ^123^I-FP-CIT SPECT SVM analysis

	Sp (%)	Sn (%)	Acc (%)	AUC	Highest beta weights	*p* value
Binary classification of PS vs CTL						
All PS vs CTL	96.7	89.5	92.9	0.97	S (more) C/P ratio (more) S-AI	< 0.001
Binary classifications of each combination of two PS						
PD vs MSAP	95 (fixed)	79.4		0.66	C (mean) C/P ratio (less) P-AI	< 0.001
	63.8	58.2	61.1			
PD vs PSP	95 (fixed)	86		0.71	C (more) C (less) P-AI	< 0.001
	61.9	66.9	64.2			
PD vs CBS	95 (fixed)	82.3		0.78	C/P ratio (more) C/P ratio (less) S-AI	< 0.001
	76.2	65.9	72.0			
MSAP vs PSP	64	64	62.9	0.64	S (less) C/P ratio (more) C-AI	0.07
MSAP vs CBS	84.6	82.6	83.7	0.95	P (mean) P (less) S-AI	< 0.001
PSP vs CBS	72.7	82.6	73.9	0.79	P (less) S (mean) S-AI	< 0.001
Binary classifications of each PS vs all other PS						
PD vs non-PD	69.9	45	58.4	0.60	C (more) C/P ratio (less) S-AI	0.01
MSA-P vs non-MSA-P	77.1	71.4	74.4	0.78	C/P ratio (more) C-AI	0.002
PSP vs non-PSP	54.2	66.7	60	0.58	C/P ratio (more) S-AI	0.06
CBS vs non-CBS	83.3	69.1	76.7	0.88	P (mean) S-AI	< 0.001

Binary classification of each combination of PS showed the best results when separating CBS from the other conditions—with Acc 83.7% against MSA-P, Acc 73.1% against PD and Acc 73.9% vs PSP. S-AI was also one of the strongest predictors. Correct classification of PD vs MSA-P and PSP revealed less robust but still satisfactory results, with Acc 63.5% and 64.8%, respectively.

When assessing binary classification of each PS vs all other PS, we observed the best results when classifying all other PS from MSA-P (Acc 74.4% with Sp 77.1% and Sn 71.4%) and from CBS (Acc 76.7% with Sp 83.3% and Sn 69.1%). On the other hand, correct classification was modest for PD (Acc 58.4%) and PSP (60%).

### Designing a tentative diagnostic flowchart

**Fig. 4:**
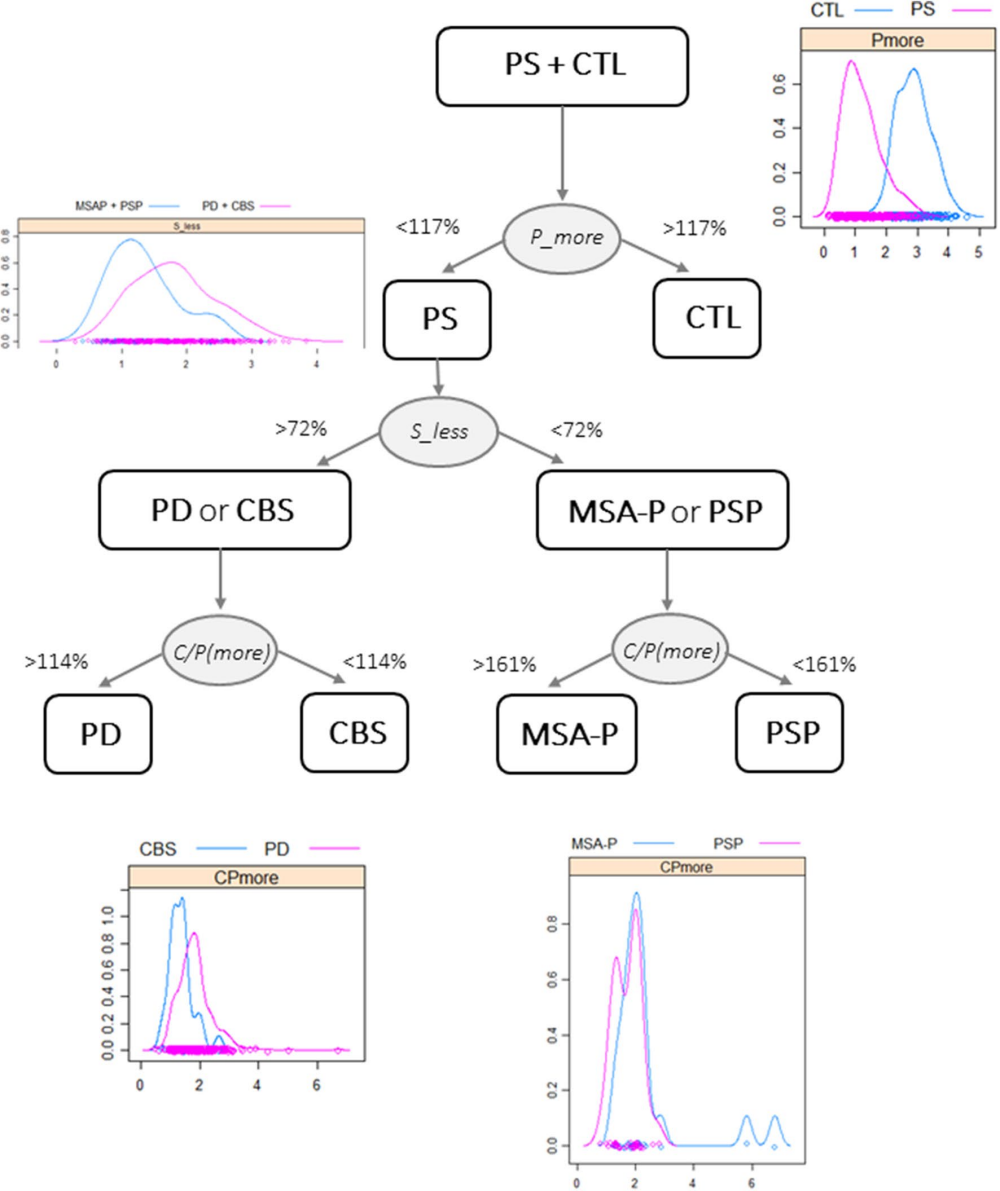
A tentative diagnostic flowchart using relevant outcomes [P(more), S(less) uptakes and C/P ratio on the more affected side] from the semiquantitative analysis to separate CTL and the various PS (PD, MSA-P, PSP or CBS) groups according to reference limits expressed as percentages of normal values. Selective density plots are shown at each step

To construct a simple diagnostic flowchart that could be used by other centers, the most informative outcomes obtained from the univariate semiquantitative BRASS analysis (Table 1) were converted into percentages, with 100% corresponding to the reference limits based on a previous work [28]. As shown on Fig. 4, a step-by-step procedure may allow to best separate (1) CTL from PS using P (more) (*p* < 0.0001); (2) PD and CBS from MSA-P and PSP patients using S (less) (*p* < 0.0001); (3) PD and CBS by means of the C/P ratio (more) (*p* < 0.0001), and (4) MSA-P and PSP also with C/P ratio (more) (*p* < 0.03). Density plots of these particularly discriminant outcomes were added at each step to illustrate how groups are set apart.

## Discussion

We here presented an SVM analysis aiming at separating degenerative parkinsonisms strictly based on their semiquantitative ^123^I-FP-CIT SPECT uptake values and related combined parameters. This analysis was tested on highly homogeneous data collected from a large, single-center cohort of subjects with well-characterized PS scanned with identical acquisition and processing imaging protocol.

Univariate statistics of semiquantitative evaluation confirm previous findings [33] of significantly decreased uptake in all forms of PS compared to subjects with non-degenerative conditions, namely lower striatal uptake, as well as higher AI and C/P ratio.

Thanks to previously established local age-dependent reference limits for striatal VOIs uptake, AIs and C/P ratio [28], we also confirmed that CBS subjects have a relative preservation of presynaptic dopamine transporters as they exhibited higher P uptake in comparison to PD (*p* < 0.005) and higher P, C and S uptake compared to MSA-P (*p* < 0.0005) and PSP (*p* < 0.001). In addition, we found significantly higher S-AI in the CBS group in comparison to PSP and lower C/P ratio than PD, MSA-P and PSP (all *p* < 0.005) [34]. PD subjects had an intermediate degree of striatal impairment with higher uptake ratio in all striatal VOIs compared to MSA-P and PSP. These results are in keeping with previous works [35].

We here report a striking SVM classification accuracy (92.9%, AUC 0.97) in disentangling PS from CTL, with a major contribution of both uptake and asymmetry parameters. Previous SVM studies have already attempted to separate PD from CTL subjects using ^123^I-FP-CIT SPECT striatal uptake and length/volume [36] or complex SPECT and biological data (including serum and CSF) from the Parkinson Progressive Markers Initiative cohort [19] with Acc 96–97%.

Furthermore, binary classification of each combination of PS allowed Acc 62.9–83.7%, with the best results obtained when separating CBS from each other PS. Considering the discussion above, these findings are not surprising, as it is well recognized that CBS harbors a specific pattern of impairment—i.e., relative striatal uptake preservation and moderate-to-high AI, especially in comparison to MSA-P (Acc in the present study 83.7%) and PSP (Acc 73.9%), which in turn exhibit a more severe and symmetrical impairment of striatal uptake. Conversely, we observed an intermediate level of uptake and AI in PD, which could explain why head-to-head classification accuracy of PD vs other PS is lower (63.5–73.1%).

In previous studies, Haller et al. observed 97% accuracy for the classification of PD subjects vs. a heterogeneous group of other PS, including mainly MSA, but also vascular parkinsonism, dementia with Lewy bodies and psychogenic parkinsonism, using fractional anisotropy DTI. Thanks to MRI atlas-based volumetry, Huppertz et al. found balanced accuracies superior to 80% for the distinction of PD from PSP and MSA, whereas a VBM study of Focke [17] found balanced accuracies superior to 80% for the distinction of PD from PSP and MSA, whereas a VBM study of Focke et al. showed 87.1% and 71.9% accuracies in separating PD from PSP and MSA, respectively [22]. Cherubini et al. were able to obtain 100% correct classification in classifying PD and PSP based on white matter atrophy. In addition, postsynaptic D2/D3 [20] were able to obtain 100% correct classification in classifying PD and PSP based on white matter atrophy. Moreover, postsynaptic D2/D3 ^18^F-desmethoxyfallypride (DMFP) PET SVM has shown 70–75% accuracy for the distinction of PD from MSA and PSP) [18]. Although these efforts are commendable, they also have inherent limitations—e.g., small sample sizes [20, 22], the debatable choice of merging several APS groups together [18], the presence of significant gray matter changes only in patients with long disease duration, when the added value as compared with clinical evaluation is limited [17] or the need for complex and time-consuming processing [19] that is not compatible with a clinical routine application.

As the diagnosis of PD and APS can be challenging when solely based on clinical evaluation, several functional imaging ligands have been developed to improve diagnostic accuracy. Using multimodal imaging, i.e., presynaptic dopamine SPECT imaging, postsynaptic raclopride PET or IBZM SPECT, and myocardial ^123^I-metaiodobenzylguanidine (MIBG) scintigraphy can be useful in distinguishing PD from APS, especially in atypical presentations and early cases. However, this requires performing several scans and exposing the patients to a significant amount of radiation, not to mention major costs for the medical facility. With the present study, we have been able to show accurate classification of PD and APS solely based on striatal semiquantitative DaT SPECT, a molecular imaging technique widely available in clinical practice and the only approved one by both 6FDA and European medical agencies for the distinction of PS from non-degenerative forms of parkinsonism and tremor. The tentative flowchart proposed on Fig. 4, although simplified and subject to many exceptions, may guide clinicians in the differential diagnosis of PS and non-degenerative forms of parkinsonism.

The strengths of our study comprise the large cohort of subjects with PS (including almost 300 subjects with PD and 90 with APS) who were scanned at a single-center level with the same acquisition and processing SPECT protocol. In addition, the semiquantitative parameters used for SVM classification are easily obtainable by any nuclear medicine facility. This could ensure replication of the present results in other centers and potential daily utilization of the highest SPECT predictors in helping to disentangle the various forms of PS. Our study also has limitations. First, diagnosis is based on clinical diagnostic criteria and most cases have not been confirmed by autopsy. However, we are confident that the retrospective nature of the study, the assessment of DaT SPECT by two unrelated visual and semiquantitative methods and a thorough follow-up by neurologists specialized in movement disorders (mean follow-up 4.4 years) should have ensured a low rate of diagnostic misattribution. Second, our CTL group is not based on healthy asymptomatic subjects, but on patients with clinically diagnosed non-degenerative forms of parkinsonism or tremor (mainly ET, DIP and PP). Again, the retrospective design of the study and the long follow-up for most cases allowed us to include only subjects whose condition did not convert into any neurodegenerative process over time and who presented a normal DaT SPECT. In addition, we were not able to obtain a clinical rating scale of motor severity (e.g., MDS-UPDRS-III) for every included subject. Using such a clinical scale as a covariate (instead of disease duration) would possibly have allowed an even better performance of SVM-based discrimination between PD and atypical conditions. Finally, the same SPECT machine and gamma camera have been used for all subjects included in the present study. This certainly helped in obtaining homogeneous uptake values. However, despite 6-month periodic quality controls ensured that scanner sensitivity was globally stable, we cannot fully exclude that time slightly affected the image quality.

In conclusion, our results indicate that semiquantitative striatal ^123^I-FP-CIT SPECT assessment provides a promising approach to distinguish reasonably well CTL, PD and APS, and that, in combination with SVM, a satisfactory classification can be obtained at the individual level. SVM and other computer-aided classification systems represent a valuable tool to assist the clinician’s daily evaluation of patients with PS.
